# SmallFishBD: An extensive image dataset of common native small fish species in Bangladesh for identification and classification

**DOI:** 10.1016/j.dib.2025.112193

**Published:** 2025-10-17

**Authors:** Md Hasanul Ferdaus, Rizvee Hassan Prito, Masud Ahmed, Syeda Raisha Abedin Ohona, Khandaker Golam Morshed, Israt Jahan Jarin, Mohammad Manzurul Islam, Nishat Tasnim Niloy, Md Sawkat Ali, Maheen Islam, Taskeed Jabid, Md Mizanur Rahoman

**Affiliations:** a Department of Computer Science and Engineering, East West University, Dhaka, Bangladesh; b Department of Agricultural Extension, Ministry of Agriculture, Bogura, Bangladesh; c Department of Computer Science and Engineering, Begum Rokeya University, Rangpur, Bangladesh

**Keywords:** Aquaculture, Computer vision, Ecological monitoring, Image processing, Machine learning, Marine Biology, Object Detection

## Abstract

This data article presents a comprehensive image dataset of ten native small fish species commonly found in Bangladesh: Bele (*Glossogobius giuris*), Chanda Nama (*Chanda nama*), Chela (*Salmostoma bacaila*), Guchi (*Mastacembelus pancalus*), Kachki (*Corica soborna*), Mola (*Amblypharyngodon mola*), Kata Phasa (*Stolephorus tri*), Pabda (*Ompok pabda*), Puti (*Puntius sophore*), and Tengra (*Mystus vittatus*). The dataset was carefully curated to facilitate the study and research in fish species identification, classification, and biodiversity monitoring. Specimens of these species were collected from various fish markets in the capital city Dhaka. Different varieties of fish are supplied to Dhaka city from diverse geographical locations in Bangladesh. Thus, the dataset ensures a representative sampling of local aquatic biodiversity.

To maintain uniformity across samples, images were captured using a smartphone camera under a standardized and controlled environment. Each specimen was placed against a neutral background with consistent lighting conditions. This limits environmental variability and enhances image quality for analytical use. The dataset contains high-resolution original images that were augmented using standard data augmentation techniques. This augmentation introduced variations such as rotations, flipping, and brightness adjustments. This expands the dataset and improves its utility for training robust machine learning (ML) and deep learning (DL) models in computer vision applications.

The dataset has significant reuse potential across multiple domains. It serves as a critical resource for researchers and industry experts to develop automated systems for fish species identification and classification, particularly in the context of the rich aquatic biodiversity in Bangladesh. Furthermore, the dataset can facilitate ecological and environmental studies and research by supporting the monitoring of native fish species distribution and population dynamics. Its structured format facilitates integration into ML/DL pipelines that can foster advancements in fisheries management, sustainable aquaculture, conservation biology, and economic and cultural studies. Thus, the dataset represents a significant step towards integrating technological advancements and ecological sustainability. This article outlines the utility of the data, the dataset structure, the data collection methodology, and the applied augmentation processes to ensure transparency and reproducibility for future research endeavors.

Specifications Table

**Table:** 

Subject	Computer Sciences, Biological Sciences, Agricultural Sciences
Specific subject area	Machine Learning and Computer Vision-based automated identification and classification of common native small fish species.
Type of data	RGB Images (raw and augmented) having a resolution of 320 × 320 in JPEG format.
Data collection	Ten common varieties of native small fish were sourced from the wholesale fish markets of Bangladesh after verifying each fish category by acquiring information from domain experts and trusted online resources. High-quality images of each procured fish specimen were captured from several angles with a smartphone camera to acquire visual knowledge of the features of different fish categories from several perspectives. Photos have been shot under a white LED light and on a neutral background. The captured 3:4 aspect ratio of images was uniformed by adding padding along the heights and all images were resized to 320 × 320 pixels to effectively utilize storage capacity without sacrificing the feature details in images. Images of each fish variety have been stored in a folder named after the most commonly used local name of that fish variety. This way our raw dataset is produced with the name *SmallFishBD*. Each image of the raw dataset has been augmented eleven (11) times to produce the augmented version of the dataset. The augmented version, called *Augmented SmallFishBD*, carries the original and the augmented images in each folder of fish varieties. The processing and augmentation of images have been conducted by utilizing several Python libraries and modules.
Data source location	The fish specimens were collected from the following regions in Bangladesh: • Dhanmondi Bazar, Dhaka, Bangladesh (23.7481° N, 90.3692° E) • Meradia Kacha Bazar, Dhaka, Bangladesh (23.7629° N, 90.4447° E) • Kalshi Bazar, Dhaka, Bangladesh (23.8217° N, 90.3744° E) • Kawran Bazar, Dhaka, Bangladesh (23.7523° N, 90.3944° E) • Rampura Bridge Fish Makret, Dhaka, Bangladesh (23.7697° N, 90.4245° E)
Data accessibility	Repository name: SmallFishBD: A Comprehensive Image Dataset of Common Small Fish Varieties in Bangladesh for Species Identification and Classification Data identification number:10.17632/8jvxtvz52x.2 Direct URL to data: https://data.mendeley.com/datasets/8jvxtvz52x/2
Related research article	*None.*

## 1 Value of the Data

• **Fish Species Identification and Taxonomy:** The dataset provides a detailed visual record of small fish species native to Bangladesh that can support researchers and taxonomists in accurately detecting and classifying different categories. It contributes to a better understanding of biodiversity and the ecological significance of various species.
• **Machine Learning and Computer Vision Research:** The dataset serves as a benchmark for evaluating machine learning and deep learning models for fish identification and classification [1]. It enables the creation of automated systems for species recognition that can be applied in fisheries for real-time monitoring and management.
• **Food Security and Aquaculture:** Small fish are essential to the diets and nutrition of millions of people in Bangladesh [2]. This dataset can support sustainable aquaculture by identifying key species, ensuring their availability, and helping to mitigate overfishing, ultimately contributing to food and nutrition security.
• **Ecological Significance and Conservation:** Native small fish play a vital role in aquatic food chains and ecosystems balance. This dataset supports monitoring of species trends, identification of vulnerable populations, and development of conservation policies. It also aids in understanding the effects of climate change, habitat degradation, and pollution, while facilitating collaborative ecological research across shared water bodies in the region.
• **Post-harvest Fish Processing:** The dataset supports the development of automated imaging systems for sorting and grading small fish, enhancing efficiency, reducing manual labor, and improving quality control in commercial fish processing.
• **Socioeconomic and Educational Applications:** Small fish varieties are integral to the cultural and economic fabric of Bangladesh. The dataset is a valuable resource for analyzing market trends and supporting livelihood development within fishing communities. It also serves as an educational tool to raise awareness among students, policymakers, and the public about the importance of aquatic biodiversity.

## 2 Background

Native small fish species are an integral part of the aquatic biodiversity in Bangladesh [3]. Bangladesh’s warm, humid, and monsoon-driven climate with abundant freshwater bodies creates ideal conditions for diverse small indigenous fish species to thrive. They play critical roles in ecosystems and local economies. Moreover, they are one of the key sources of nutrition for millions of people in the country [2]. Despite their ecological and economic significance, comprehensive digital documentation has not been prepared for such species. As a result, their lack of accessibility hinders opportunities for research, conservation, and technological applications. Moreover, the scarcity of species-specific data previously led to misidentification, overfishing, poor conservation planning, and inaccuracies in trade and biodiversity assessments. The referenced datasets [[4], [5], [6]] provide valuable freshwater fish images captured in natural settings, though they also exhibit variations in lighting, background elements, image dimensions, and clarity, with limited augmentation and folder-level diversity that may influence research applications. Our *SmallFishBD* dataset contains images of small and native fish species of Bangladesh (Bele, Nama Chanda, Chela, Guchi, Kachki, Mola, Kata Phasa, Pabda, and Tengra) featuring high-quality visual data resources that can facilitate the identification and classification of native small fish species commonly found in Bangladesh through computer vision technologies, such as machine learning and deep learning models [7]. Images in this dataset are sharp and contain controlled background and lighting, and uniform dimensions, making it ideal for computer vision-based applications. Its prospective utility spans diverse real-world applications, such as sustainable fisheries management, aquaculture optimization, ecological research, and food supply chain transparency.

The data was collected following the established methodologies for constructing image datasets for computer vision applications. Specimens of ten native and most commonly found species were procured from different fish markets located in various places of the capital city, Dhaka to ensure diversity, representativeness, and fairness of selection. A high-definition smartphone camera was used to capture images in a controlled environment to maintain uniformity and consistency and adhere to the best practices in image dataset creation. To expand the dataset and enhance its applicability in training machine learning models, standard data augmentation techniques were applied to the dataset so that it can represent real-world variations and improve model generalizability for automated fish species identification and classification.

## 3 Data Description

**Fig. 1:**
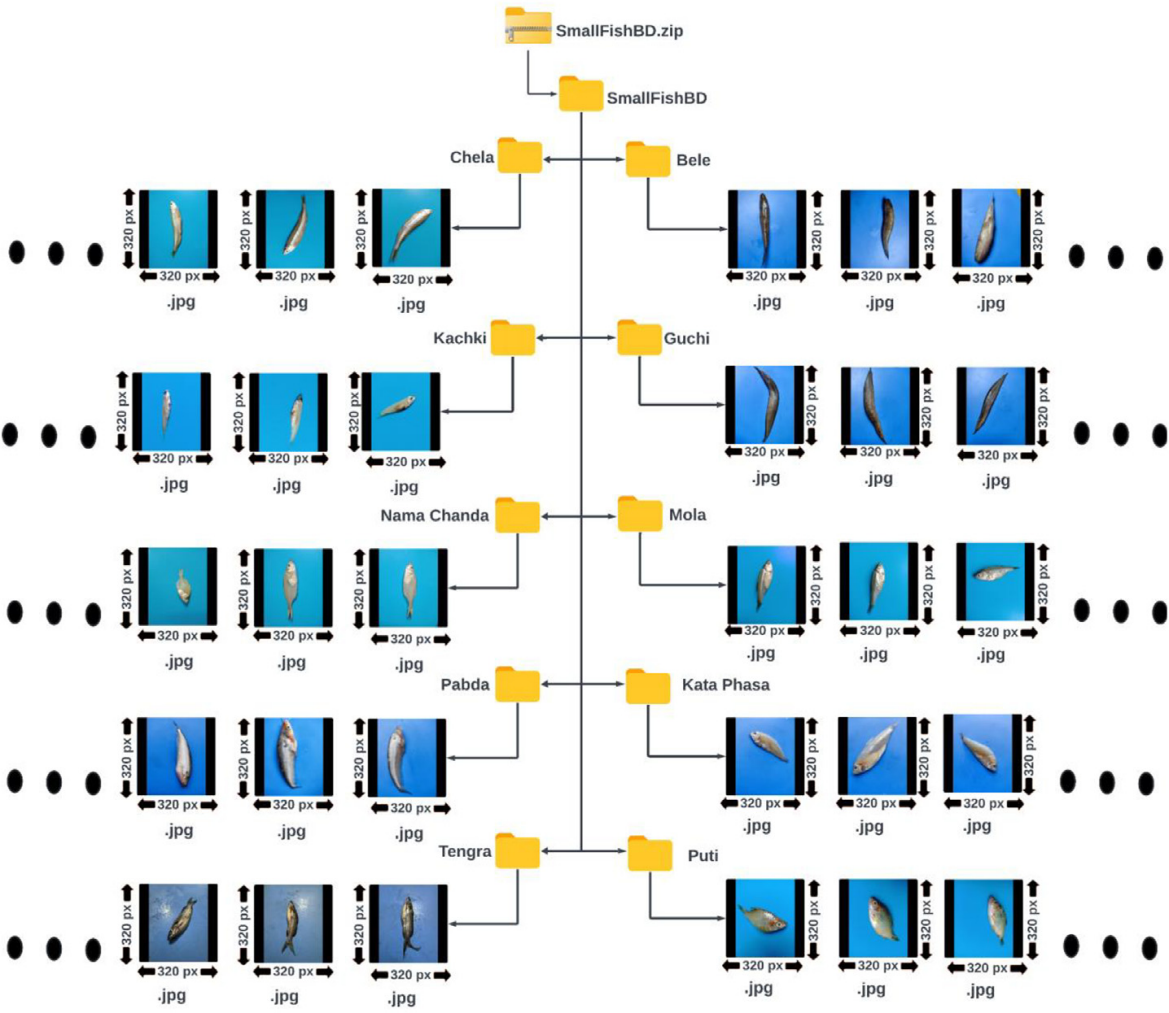
Folder structure of the original SmallFishBD dataset.

Our image dataset of ten (10) varieties of native small fishes has been orchestrated by partitioning into two sets, *SmallFishBD* and *Augmented SmallFishBD*. Each set is represented in a folder named after the partition name. Ten subfolders are included in each of these main folders, labeled with the most commonly used local Bengali name of each fish variety. Each subfolder contains the corresponding fish images in JPEG format with a resolution of 320 × 320 pixels. Images of the *SmallFishBD* dataset are presented with black padding along the height to maintain an aspect ratio of 1:1 (i.e., to ensure the height and the width of the images are equal). The *Augmented SmallFishBD* folder contains eleven (11) augmented images for each original image of the *SmallFishBD* dataset, along with the original images from the *SmallFishBD* dataset. Both datasets are published in compressed ZIP format to utilize storage capacity effectively. Detailed information on our dataset is presented in Table 1. The folder structure of our original datasets (*SmallFishBD*) is illustrated in Fig. 1. The augmented dataset folder (*Augmented SmallFishBD*) also follows a similar folder structure. Table 2 shows brief biological and morphological details for each fish species in our dataset.

**Table 1:** Dataset description with fish species, image count, and dataset size.

Fish Varieties (Bengal Names)	Number of images in the *SmallFishBD* dataset		Original size and compressed size of the *SmallFishBD* dataset	Number of images in the *Augmented SmallFishBD*		Original size and compressed size of the *Augmented SmallFishBD* dataset
*Bele*	205		Original Size= 36.2 MB Compressed Size= 28.4 MB	2460		Original Size= 617 MB Compressed Size= 527 MB
*Chela*	190			2280		
*Guchi*	164			1968		
*Kachki*	247			2964		
*Mola*	179			2148		
*Nama Chanda*	110			1320		
*Kata Phasa*	129			1548		
*Pabda*	125			1500		
*Puti*	218			2616		
*Tengra*	133			1596		
	**Total**	1700		**Total**	20,400

**Table 2:** Biological and Morphological details of the fish species.

Fish Species	Morphological Details	Sample Captured Images
Bengali Name: Puti **Common Name:** Spot-fin Swamp Barb Scientific Name: *Puntius sophore* **Other Local Name:** Punti, Jati Punti, and Vadi Punti	Mark: A black spot at the start of the tail and the origin place of the dorsal fin. Body: Fairly squeezed. Outline: The dorsal one is more convex than the abdomen. Mouth: Small, closing, and the upper jaw is a little extended. Fin: The length of the pectoral fin is the same as the head without the snout portion. Barbels: Absent. Color: Silvery, the backside is gray-greenish or brownish. White in the under part of the body [8].	
Bengali Name: Mola **Common Name:** Mola Carplet, Pale Carplet Scientific Name: *Amblypharyngodon mola* **Other Local Name:** Moua and Moah	Mark: Dark spot at dorsal and anal fins. Body: Compressed from the sides. Outline: The dorsal side is more convex than the ventral side. Mouth: Positioned at the front of the head and angled slightly upward. Fin: The caudal fin is highly divided and has pointed lobes. Barbels: Absent. Color: The back is light greenish, but the side and below parts are silvery [8].	
Bengali Name: Chela **Common Name:** Large Razorbelly Minnow Scientific Name: *Salmostoma bacaila* **Other Local Name:** Narkeli Chela	Mark: None Body: Prolonged and highly pressed from two sides. Outline: The Dorsal side is slightly more convex than the ventral side. Mouth: Inclined. The lower jaw has a prominently developed symphysial knob. Fin: Dorsal fin is ended long before the anal fin. Barbels: Absent. Color: The dorsal portion is blackish, but the body is silvery [8].	
Bengal Name: Nama Chanda Common Name: Elongate Glass Perchlet Scientific Name: *Chanda nama* **Other Local Name:** Chanda	Mark: Dark mark at the start of the anal fin, countless black dots in some parts of the body. Body: Highly compressed and nearly flat from the side. Outline: Dorsal and ventral sides are almost identically convex. Mouth: The Lower jaw is longer compared to the upper jaw. Fin: Has orange and black pronged caudal fin. Barbels: Absent. Color: Yellowish translucent white. The head of the first and second dorsal fins is black [8].	
Bengal Name: Guchi **Common Name:** Striped Spiny Eel Scientific Name: *Mastacembelus pancalus* **Other Local Name:** Pankal, Turi, and Chirka	Mark: The upper part of the body and the top to middle part of both sides of the body contain numerous dark spots. Body: Long and compressed. Outline: Eel-like elongated outline. Mouth: Mouth is subterminal, and the snout is conical. Fins: Dorsal and anal fins are prolonged and unattached to the Caudal fin which is tiny and round. Barbels: Absent Color: The top is black-yellowish and the beneath portrays yellow-whitish color. The upper part of the body and the top to middle part of both sides of the body contain numerous dark spots [8].	
Bengal Name: Bele **Common Name:** Tank Goby Scientific Name: *Glossogobius giuris* **Other Local Name:** Bailla and Balia	Mark: Often shows small dark spots. Body: Elongated, cylindrical towards the front, and compressed from behind. Outline: The dorsal side is more convex than the ventral side. Mouth: Little skewed, upper jaw is shorter than lower jaw, dense lips. Fins: Anal fin and the second dorsal fin are posteriorly oriented. The caudal fin is edgy or mildly rounded. A small gap lies between the two dorsal fins. Barbels: Absent. Color: Blackish green or olive green in the upper body, yellowish in the caudal and dorsal fins, and whitish in the pelvic and anal areas [8].	
Bengal Name: Pabda **Common Name:** Pabdah Catfish Scientific Name: *Ompok pabda* **Other Local Name:** Madhu Pabda	Mark: A dark spot is located along the lateral line and above the pectoral fins. Body: Long and pressed at the sides, the head is flattened in a dorso-ventral direction, rounded snout. Outline: Dorsal and ventral sides are gently convex. Mouth: Lower jaw longer. Fins: The dorsal fin appears on top of the ending half of the pectoral. The caudal fin is pronged with circular lobes facing downwards. Barbels: Two pairs. The maxillary barbels reach at the end of the pectoral fin or the midpoint of the anal fin. Color: Silvery-grey; black on top; belly is light white [8].	
Bengal Name: *Tengra* **Common Name:** Striped Dwarf Catfish Scientific Name: *Mystus vittatus* **Other Local Name:** Guitta Tengra	Mark: It contains black stripes, and a deep spot is present on the shoulder. Body: Long and a little compressed. Outline: A slightly arched dorsal profile and a flatter ventral side. Mouth: Small and terminal. Fin: Short adipose fin, ended up far beyond rayed dorsal fin but before the anal fin. Barbels: Four pairs. The maxillary barbels reach past the pelvic fin, sometimes to the end of the anal fin. Color: Soft gray-silver, about 5 longitudinal deep black lines running along the sides., and a deep spot is present on the shoulder [8].	
Bengal Name: *Kachki* **Common Name:** Ganges River Sprat Scientific Name: *Corica soborna* **Other Common Name:** Guramach and Taki	Mark: A dim lateral stripe goes longitudinally, black spot can be seen in the caudal fin. Body: Long and compressed laterally. Outline: A nearly straight dorsal profile and a slightly curved ventral side. Mouth: Terminal, moderate size, and has very small or no teeth in the jaws. Fins: A detached finlet originated from the last two anal fin rays. The caudal fin is pronged, and the lower lobes are a bit lengthy. Barbels: Absent. Color: Silvery or brownish. A dim lateral stripe goes longitudinally, black spot can be seen in the caudal fin [8].	
Bengal Name: *Kata Phasa* **Common Name:** Spined Anchovy Scientific Name: *Stolephorus tri* **Other Local Name:** Kata Phaysha	Mark: A vivid silvery lateral stripe goes longitudinally. Body: Elongated and a bit compressed laterally. The snout is outwardly extended and slightly pointed. Outline: The dorsal and ventral sides are disproportionately convex. Mouth: Slightly superior, snout is protruding, and the tip of the upper jaw (maxilla) is pointed. Fins: Have small dorsal, pelvic, and pectoral fins. Between the pectoral and pelvic fins, 6 to 8 spikes similar to needles appear. A rare spike arises on the pelvic scute oriented between the bases of the fins. Barbels: Absent. Color: Silvery. A vivid silvery lateral stripe goes longitudinally [9].	

**Fig. 2:**
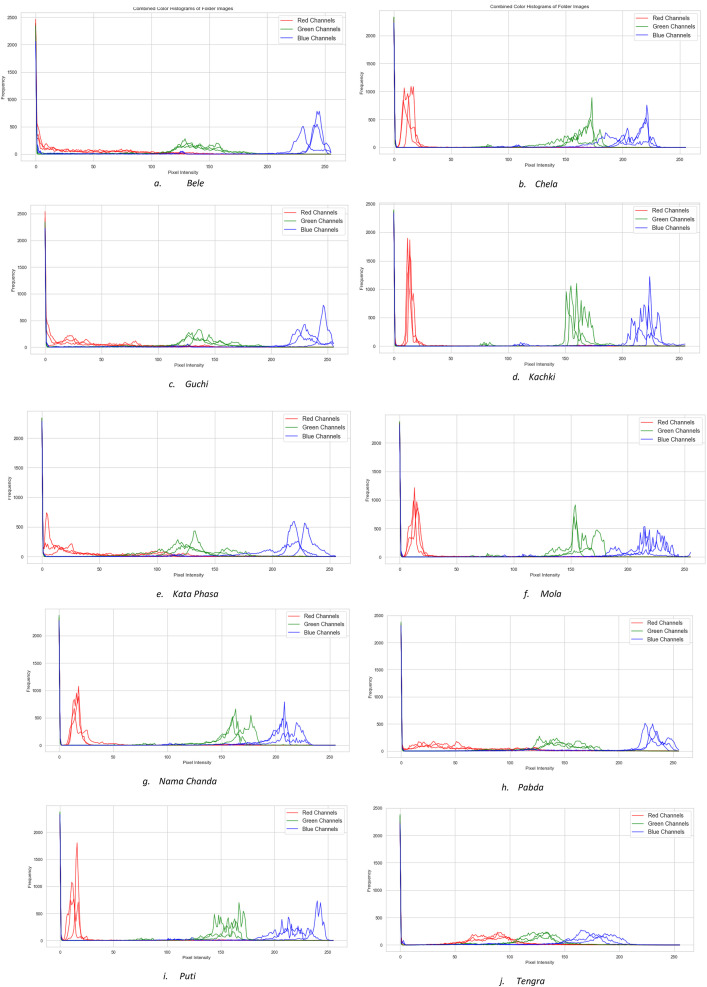
Combined histogram plots of three images from each fish species.

To indicate the variations in intra-class images in the dataset, we demonstrated combined histograms of three randomly chosen images from each fish species in Fig. 2. Each plot has three-pixel intensity distributions for red, green, and blue channels, visualizing the similarities and variations in pixel densities among the RGB channels across three images.

## 4 Experimental Design, Materials and Methods

**Fig. 3:**
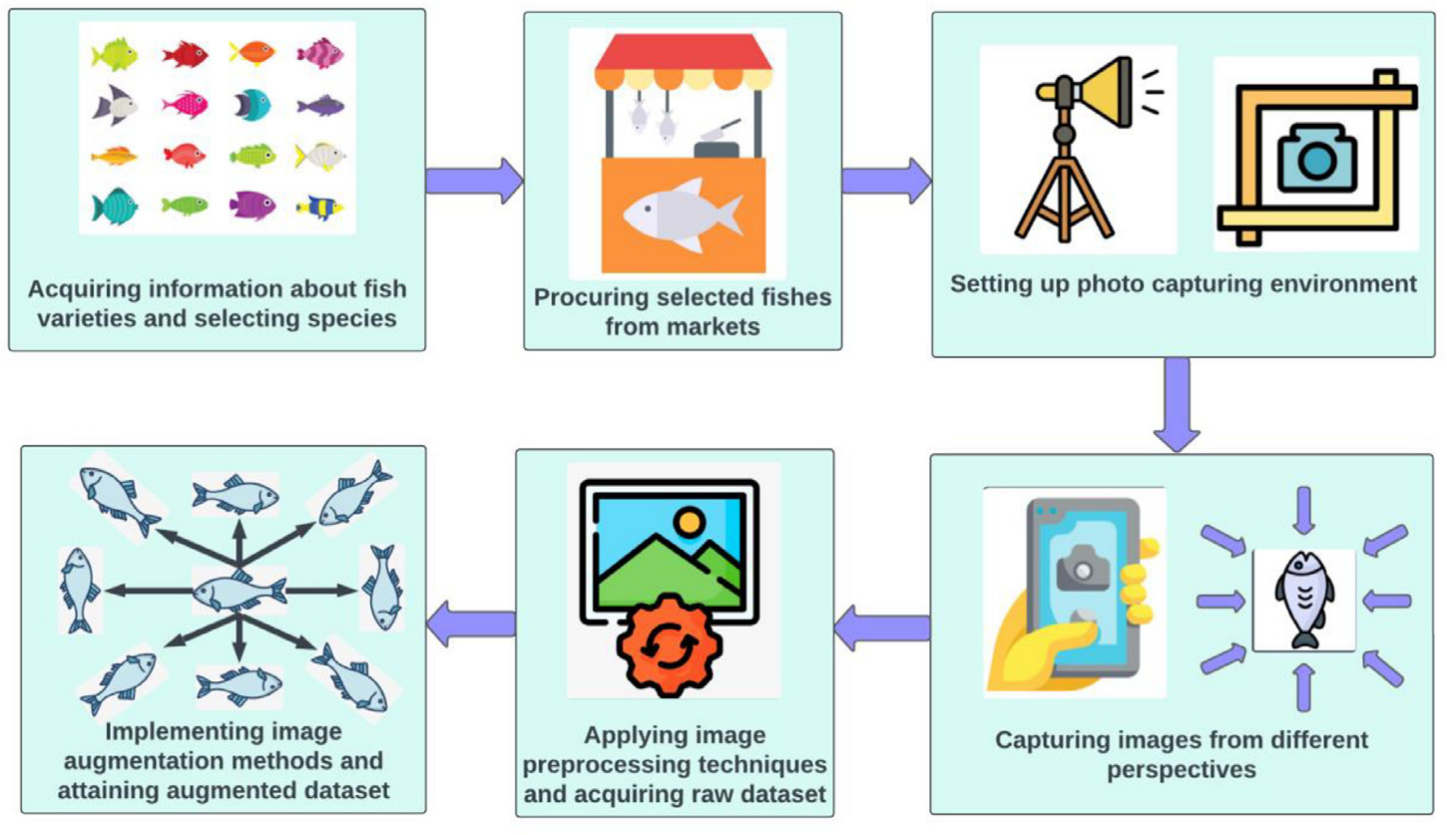
Steps followed in the dataset construction process.

Our formulated dataset is crafted following a systematic approach, which is illustrated in Fig. 3. Such measures are pursued based on previous experiments on devising similar visual resources.

Ample knowledge of fish species is paramount in fabricating datasets on fish varieties. Comprehensive research was conducted to obtain information on available small fish varieties in the markets before procuring the fish specimens. Adequate details on fish categories were gained by relying on trusted online resources, where scientific articles on small fishes in Bangladesh were incorporated as references. Moreover, the domain expert’s opinion has assisted us with the information for a thorough inspection of the features of each kind of fish since different categories of fish may present similar characteristics. After completion of the small fish species selection, fresh fish specimens were purchased from different fish markets located in geographically distributed places of Dhaka city in Bangladesh. Dhaka, being the capital city of the country, attracts fish supplies from various geographical locations of Bangladesh. Fish specimens with visible deformities were excluded.

To photograph the fish with appropriate light and background, we harnessed a place with white LED light and a blue background to ensure color-neutral contrast with the fish specimens. White LED light has facilitated the proper manifestation of the genuine color and texture of the fish bodies and a controlled, neutral background has averted background noise and minimized light reflection in the images. After establishing the photo shooting background, each fish from our purchased fish pool was selected once for photographing and placed at the center of the background. High-resolution images have been taken with a smartphone camera from different angles to capture the physical properties of fish from various perspectives. A uniform distance of 18 cm between the camera and fish species is maintained. The specifications of the smartphone camera are listed in Table 3. Through this image-capturing approach, we ensured diversification in our dataset. High-quality photos of different dimensions with the 2:3 aspect ratio were shot. Images were then transferred to a computer and organized into folders labeled with the names of the fish varieties.

**Table 3:** Specifications of the smartphone camera.

Camera Feature	Feature Specification
Smartphone model	Vivo V21
Resolution	64 MP (wide)
Aperture	f/1.8
Focal length	26 mm
Sensor size	1/1.72 inches
Pixel size on the sensor	0.8 µm
sensitivity to light	ISO 100–3200

**Fig. 4:**
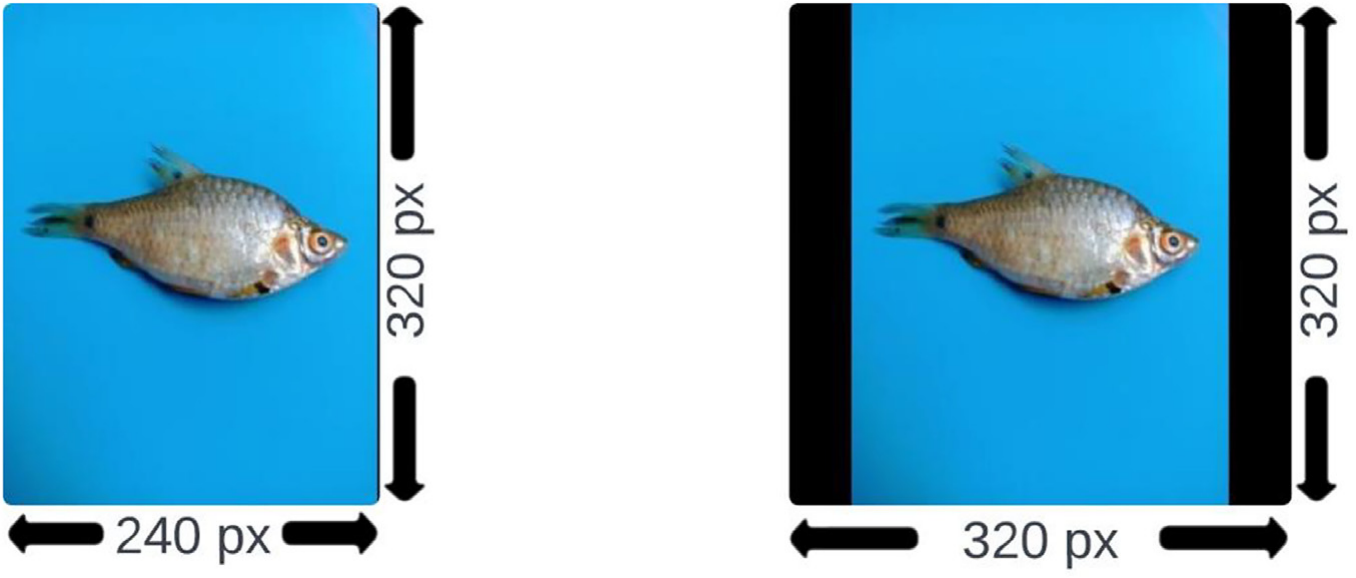
Visual comparison of a sample original image (left) with a 2:3 aspect ratio and the padded image (right) with a 1:1 aspect ratio.

Afterward, the images were resized to 240 × 320 pixels to equip the ML/DL models on the dataset without requiring high-performance computational resources. Resizing has been done utilizing the ‘Image’ module of the Python library [10]. As all the convolutional neural network (CNN) models of deep learning operate with images of uniform dimensions in height and width, our raw images are padded with black portions on the right and left sides of images to convert their aspect ratio from 2:3 to 1:1, resulting in dimensions of 320 × 320 pixels. The process of equalizing the height and width measurement is conducted by implementing a Python script of the ‘Image’ and ‘ImageOps’ modules of the ‘PIL’ library [10]. This is conducted with extreme care so that the shape of the fish within the processed images is not altered. Resizing images to a uniform dimension ensures consistent input shape for machine learning and deep learning models, facilitating efficient training and reducing computational complexity. It also helps preserve spatial relationships and enables the models to learn relevant features more effectively, improving accuracy in fish identification and classification tasks. The difference between the original images and the padded images is shown in Fig. 4. After completing all these procedures, we obtained the raw dataset consisting of 1700 images of ten small fish species of Bangladesh and published it as a compressed ZIP file (*SmallFishBD.zip)* on Mendeley Data [11]. The percentage of fish pixels to all image pixels ranges from 0.74 % to 15.73 % across all the images. This is calculated by annotating the fish part of an image in a polygon format. Later, the coordinates of the polygon points are utilized to implement the Shoelace formula (a.k.a. Gauss's area formula) using a Python script [10].

**Fig. 5:**
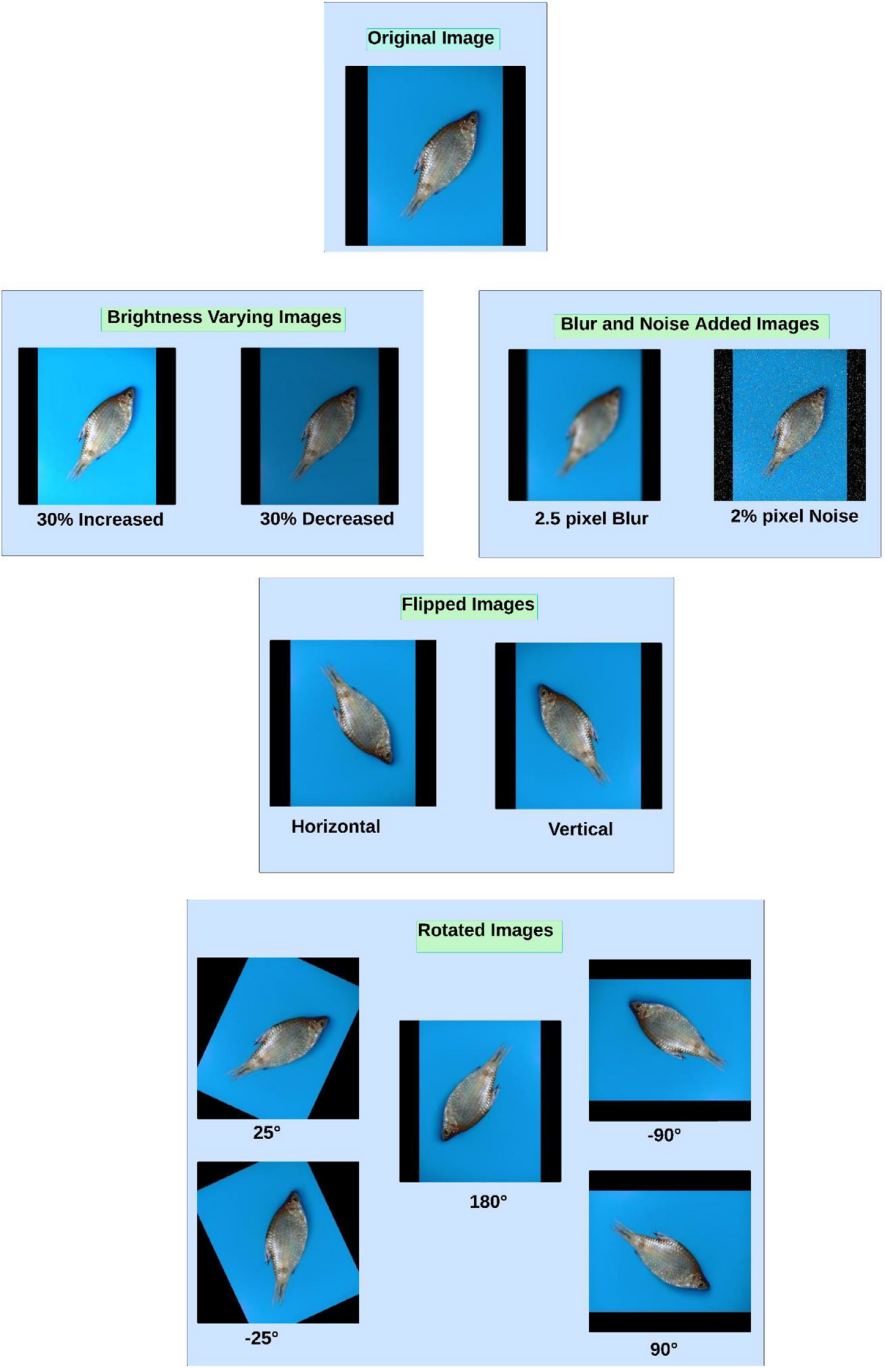
Sample images for each augmentation method applied to the dataset.

Augmentation techniques unveil enhancement in datasets, producing variegated replications of the original data depending on real-life scenarios. We have offered an augmented version of the raw dataset to perceive diversity in the dataset and provide generalization ability to the ML/DL models instructed on our augmented dataset to avert the overfitting problems in recognizing the accurate fish varieties while learning visual details of fishes from various aspects. Diversifications have been introduced to the raw dataset by incorporating 11 augmentation strategies. Afterwards, the augmented dataset is comprised of the original images and the 11 augmented versions of each original image. These augmented versions are generated utilizing a Python script importing ‘ImageEnhance’ and ‘ImageFilter’ libraries [10], resulting in a dataset of 20,400 images of the ten small fish varieties. The intention of applying each augmentation method is briefly described below. A sample image resulting from each augmentation is depicted in Fig. 5. The augmented dataset is also published as a compressed ZIP file (*Augmented SmallFishBD.zip*) on Mendeley Data [11].

**Horizontal and Vertical Flipping:** A fish specimen may be positioned in an image in various orientations, which can be vertically or horizontally flipped. To train the ML/DL models without making them dependent on any specific flipped orientation of the object, horizontal and vertical flipping variations have been introduced in our augmented dataset.

**Rotating** ±**25°,** ±**90°, 180°:** A photo can be taken by rotating the camera at various angles and a fish in an image can appear in different rotations. Incorporating ±25°, ±90°, and 180° rotational variations in the dataset will guide the ML/DL models to be independent of certain rotations.

**Brightness Variation of** ±**30** **%:** Images can be captured in brighter or darker environments. Producing one picture by enhancing brightness by 30 % and generating another by decreasing the brightness by 30 % from the original photo and training the ML/DL algorithms on these images will allow them to be robust in different brightness conditions.

**Adding 2.5 Pixel Blur and 2** **% Pixel Noise:** Adding 2.5-pixel Gaussian blur to the original images will aid the ML/DL classification models in learning the features of objects from unfocused images. A 2 % noise addition has been performed by incorporating salt and pepper noise in the original images to make the models insensitive to camera artifacts.

We have employed three renowned CNN models of various sizes and parameter counts to investigate the performance of deep learning models in classifying the small fish categories from our proposed dataset. Each of these three models (EfficientNetV2B0, MobileNetV2, and Xception) is comprised of several unique features, allowing them to identify various kinds of objects from images accurately. EfficientNetV2B0 incorporates compound model scaling, integrates fused convolution layers with MBConv convolutional network, and utilizes speed and accuracy-optimizing techniques such as neural architecture search (NAS), empowering the model to achieve higher accuracy with faster training and progressive learning quality in categorizing images across diverse datasets. MobileNetV2 is built upon inverted residuals with linear bottlenecks to extract feature representations of complex patterns without costing pictorial information and computational resources. The depthwise separable convolution structure of MobileNetV2 architecture minimizes its storage quantity and computational complexity while preserving the model’s learning competency. The Xception model takes advantage of depthwise separable convolutions within a fully convolutional architecture, leading to the acquisition of effective relational information between the spatial and channel-wise convolved feature maps, resulting in enhanced classification accuracy and reduced computational overhead. Table 4 demonstrates the implemented model size and its parameter amount.

**Table 4:** Size and total number of parameters employed in the evaluated models.

Model Name	Size	Total Number of Parameters (Millions)
EfficientNetV2B0	22.63 MB	5.93 Millions
MobileNetV2	8.66 MB	2.27 Millions
Xception	79.66 MB	20.88 Millions

The original dataset is separated into training, validation, and testing sets by maintaining a partitioning ratio of 70:15:15, respectively. Images of all sets are resized to 200 × 200 × 3, and three previously mentioned augmentation methods are selected randomly to augment the training set. Selected hyperparameters are equally applied to train all the models. Hyperparameters and their assigned values are illustrated in Table 5. Leveraging the transfer learning procedure, pre-trained weights of ImageNet are harnessed to initialize the models’ training, enabling them to accelerate convergence in the training period.

**Table 5:** Selected hyperparameters for all the applied models.

Hyperparameter Name	Value
Learning rate	0.0001
Optimizer	Adam
Loss Function	Sparse Categorical Crossentropy
Batch Size	8
Epoch	30
Early Stopping Parameters	Patience = 10 Monitor = Validation loss Mode = Minimum Restore_best_weights = True
Final Layer Activation Function	Softmax
Dropout Rate	30 %

**Fig. 6:**
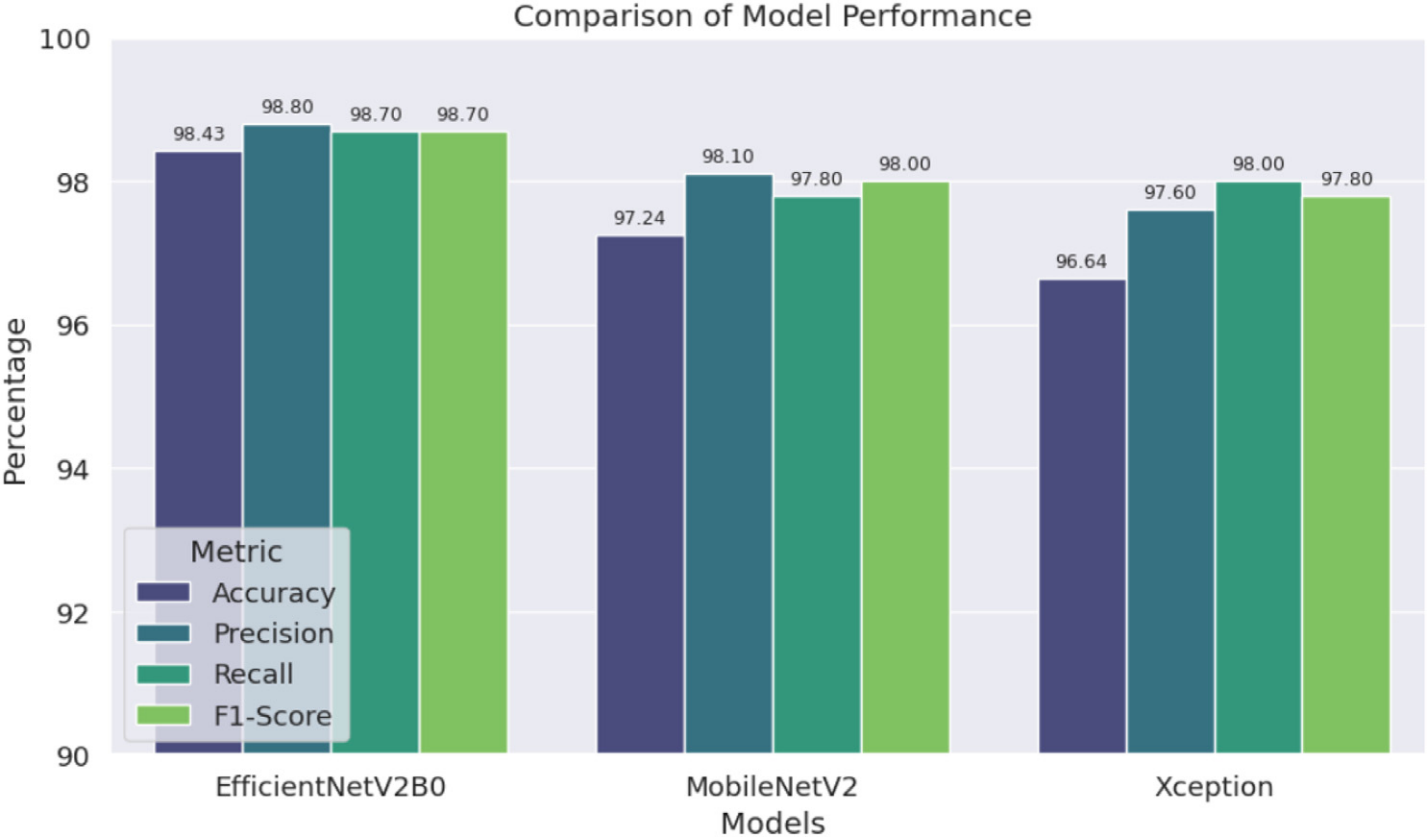
Performance (Accuracy, Precision, Recall, and F1 Score) of the models on the test dataset.

**Fig. 7:**
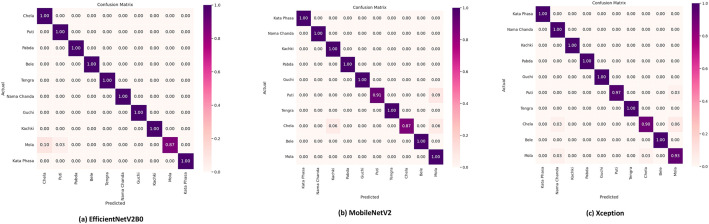
Normalized confusion matrixes of the evaluated models.

To avoid the overfitting complexity, we have exploited early stopping hyperparameters and a single dropout layer. The output of the dropout layer is transferred to the Global Average Pooling (GAP) layer, which is added to shrink the spatial dimension of the feature maps before passing them to the final layer. The final layer is included with a dense layer consisting of 10 nodes corresponding to our 10 small fish classes and utilized with the softmax activation function. The performance of the three trained models is tested rigorously on the unseen test set and evaluated with the four most effective classification metrics: Accuracy, Precision, Recall, and F1-score. In all the metrics, EfficientNetV2B0 excelled over other models by scoring 98.43 % accuracy, 98.80 % precision, 98.70 % recall, and 98.70 % F1-score, highlighting its exceptional performance in congruously identifying all types of small fishes from our proposed dataset. The evaluation results of all three models are demonstrated in Fig. 6. Models' performance in identifying each fish species is measured by Precision, Recall, and F1-score, as shown in Table 6, where the EfficientNetV2B0 model demonstrates the best performance by achieving 100 % in all performance metrics for most of the fish classes. A normalized version of the confusion matrix of each model is depicted in Fig. 7, which demonstrates the EfficientV2B0 model’s outstanding performance with 100 % accurate prediction on maximum fish classes.

**Table 6:** Performance results of the models for each fish category.

Fish Species	EfficientNetV2B0			MobileNetV2			Xception		
	Precision	Recall	F1-score	precision	Recall	F1-score	Precision	Recall	F1-score
Bele	100 %	100 %	100 %	100 %	100 %	100 %	100 %	100 %	100 %
Chela	91 %	100 %	95 %	100 %	100 %	100 %	100 %	100 %	100 %
Guchi	100 %	100 %	100 %	100 %	100 %	100 %	100 %	100 %	100 %
Kachki	100 %	100 %	100 %	100 %	87 %	93 %	97 %	90 %	93 %
Kata Phasa	100 %	100 %	100 %	86 %	100 %	92 %	90 %	93 %	92 %
Mola	100 %	87 %	93 %	100 %	100 %	100 %	100 %	100 %	100 %
Nama Chanda	100 %	100 %	100 %	100 %	91 %	95 %	100 %	97 %	99 %
Pabda	100 %	100 %	100 %	95 %	100 %	98 %	100 %	100 %	100 %
Puti	97 %	100 %	99 %	100 %	100 %	100 %	89 %	100 %	94 %
Tengra	100 %	100 %	100 %	100 %	100 %	100 %	100 %	100 %	100 %

Beyond fish classification, this dataset can support broader applications such as conservation planning by helping monitor species distribution and identifying at-risk populations. It can be integrated into educational tools to teach students and communities about local biodiversity and sustainable fishing practices. Additionally, the dataset can power mobile applications for real-time fish recognition at local markets, aiding consumers, vendors, and regulators in species verification and promoting transparency in fish trading. Such apps can also help prevent mislabeling and support informed decision-making. These extended applications enhance the dataset’s value across scientific, environmental, commercial, and educational sectors in Bangladesh and similar regions.

Future directions may include annotating the dataset for object detection and segmentation tasks, expanding species diversity to cover more native fish, and capturing images under varied environmental conditions to improve model generalization. Integrating the dataset into industry-level applications, such as automated systems for sorting, quality control, and traceability in fish markets, can significantly boost its real-world utility. Moreover, collaborations with fisheries departments and research institutions can support the development of policy tools for biodiversity monitoring and sustainable fisheries management. The dataset can also be extended to include temporal variations in morphology for seasonal analysis. These initiatives would collectively enhance the impact and scope of the research.

## Limitations

• The dataset may not fully capture the morphological variations in fish that may occur to some species due to seasonal changes, such as changes in size, color, or body features influenced by breeding cycles or environmental conditions. This limitation could affect the ML/DL model’s ability to generalize to fish collected throughout the year. Having said that, this limitation can be addressed through the incorporation of adaptive learning techniques or transfer learning using models trained on more diverse datasets when available.
• There are some differences in the number of images across the ten species due to variations in availability or market representation. Such class imbalance could introduce bias and affect the performance of models in identifying underrepresented fish species. However, this issue can be resolved via techniques such as the oversampling of minority classes or class-weight adjustments during model training without requiring additional data.
• The dataset includes diverse images captured from multiple angles under consistent lighting, with minimal occlusion and clutter. While controlled settings enhance clarity, limited variation in lighting and background may affect generalizability, guiding researchers to consider domain adaptation for broader application. Machine learning and deep learning models trained on this dataset may face reduced accuracy on wild-caught fish or under varied lighting and backgrounds due to domain shift. Such shifts can alter visual features, affecting model generalization. Acknowledging this helps set realistic expectations and highlights the need for domain adaptation or fine-tuning when applying models beyond the dataset's controlled setting.

## Ethics Statement

The authors have read and followed the ethical requirements for publication in Data in Brief and confirm that the current work does not involve human subjects, animal experiments, or any data collected from social media platforms. The fish were already deceased at the time of specimen purchase from the fish markets and ethical standards for biological specimen handling were maintained with respectful treatment. Proper handling and imaging practices were followed to avoid depictions that could be viewed as disrespectful or sensationalized.

## Credit Author Statement

**Md Hasanul Ferdaus:** Conceptualization**,** Formal Analysis, Validation, Writing - Review & Editing, Supervision. **Rizvee Hassan Prito:** Conceptualization, Investigation, Data Curation, Visualization, Writing – Original Draft. **Masud Ahmed:** Validation, Resources, Investigation, Writing – Review & Editing. **Syeda Raisha Abedin Ohona:** Data Curation, Validation, Writing - Review & Editing. **Khandaker Golam Morshed**: Data Curation, Resources. **Israt Jahan Jarin:** Data Curation, Resources. **Mohammad Manzurul Islam:** Data Curation, Resources. **Nishat Tasnim Niloy:** Data Curation, Resources. **Md Sawkat Ali:** Supervision, Project Administration. **Maheen Islam:** Conceptualization, Supervision. **Md Mizanur Rahoman:** Resources, Supervision, Project Administration.

## Data Availability

Mendeley DataSmallFishBD: A Comprehensive Image Dataset of Common Small Fish Varieties in Bangladesh for Species Identification and Classification (Original data)
